# THE VALUE OF US: EXPRESSIONS OF TOGETHERNESS IN COUPLES WHERE ONE SPOUSE HAS DEMENTIA

**DOI:** 10.1093/geroni/igz038.2445

**Published:** 2019-11-08

**Authors:** Anna Swall, Christine Williams, Lena Marmstål Hammar

**Affiliations:** 1 School of Education, Faculty of Health Sciences, School of Health and Social Studies, Falun, Sweden., Falun, Sweden, Sweden; 2 Christine E Lynn College of Nursing, Florida Atlantic University, Boca Raton, Florida, United States; 3 School of Education, Health and Society, Falun. Sweden, Karolinska Institute, Division of Nursing, Department of Neurobiology, Care Sciences, Stockholm, Sweden, Falun, Sweden

## Abstract

Background: Living with dementia involves both illness and health and involves self-care and care by others. As most persons with dementia are living in their ordinary housing, dementia affects not only the person with the disease, but also the life of the family, commonly the partner. Research show that spouse carers feel like they are losing their partners due to an inability to share thoughts, feelings and experiences as a couple. Aim: The aim of the study was to describe spouse’s experience of their togetherness when one spouse has dementia. Method: The sample consisted of eighteen recorded conversations between 15 persons with dementia and their spouses. The filmed conversations were transcribed verbatim and then analyzed using qualitative content analysis. Findings: One overarching theme arose from the data “Dementia preserved and challenged the value of “us”. Being a couple trying to preserve a sense of togetherness and have the relationship they wished for could be seen as a challenge when one spouse was living with dementia. Conclusion: Based on our results, we suggest that practitioners should help couples to reinforce or strengthen their bonds as a couple to maintain well-being. Future studies should examine couplehood under differing conditions such as long versus short term relationships. Prior relationship quality may also be a factor influencing the sense of couplehood following a serious health challenge such as dementia.

